# RrTTG1 promotes fruit prickle development through an MBW complex in *Rosa roxburghii*

**DOI:** 10.3389/fpls.2022.939270

**Published:** 2022-08-29

**Authors:** Xiaolong Huang, Peipei Yi, Yanjing Liu, Qiaohong Li, Yu Jiang, Yin Yi, Huiqing Yan

**Affiliations:** ^1^School of Life Sciences, Guizhou Normal University, Guiyang, China; ^2^Key Laboratory of Plant Physiology and Development Regulation, Guizhou Normal University, Guiyang, China; ^3^Key Laboratory of National Forestry and Grassland Administration on Biodiversity Conservation in Karst Mountainous Areas of Southwestern China, Guizhou Normal University, Guiyang, China; ^4^Sichuan Provincial Academy of Natural Resource Science, Chengdu, China

**Keywords:** *Rosa roxburghii*, RrTTG1, fruit prickle, trichome, MBW complex

## Abstract

Fruit prickles are widely distributed on the pericarp and exhibit polymorphic traits at different developmental stages. Although they are multicellular appendages that are well-known for helping plants defend against biotic and abiotic stresses, their origination and molecular mechanism are still less known. Here, we studied the origination and molecular mechanism of fruit prickles in *Rosa roxburghii*. Using morphological and histological observations, we found that the fruit prickle primordium of *R. roxburghii* originated from the ground meristem that underwent cell division to form flagelliform prickles, continued to enlarge, and finally lignified to form mature fruit prickles. We amplified a homolog of candidate gene *TRANSPARENT TESTA GLABRA1* (*TTG1*) from *R. roxburghii*, named *RrTTG1*. RrTTG1 harbored four conserved WD-repeat domains and was exclusively nuclear-localized. Using qRT-PCR and *in situ* hybridization, we found that *RrTTG1* was constitutively expressed and highly expressed during the initiation and cell expansion phases of fruit prickles. Ectopic expression analysis in *Arabidopsis* proved that *RrTTG1* substantially enhanced the number of trichome and pigmentation production and inhibited root hair formation. Besides, *RrTTG1* complemented the phenotypes of the *ttg1* mutant in *Arabidopsis*, thus indicating that *RrTTG1* played pleiotropic roles akin to *AtTTG1*. We demonstrated that the RrTTG1 only interacted with RrEGL3, a homolog of ENHANCER OF GLABRA3 (EGL3), *via* yeast two-hybrid (Y2H) and bimolecular fluorescence complementation (BiFC) assays. Briefly, RrTTG1 might positively regulate the initiation of fruit prickle primordium and cell enlargement by forming the RrTTG1-RrEGL3-RrGL1 complex in *R. roxburghii*. Therefore, our results help characterize the *RrTTG1* in *R. roxburghii* and also elucidate the establishment of the prickles regulatory system in the Rosaceae plants.

## Introduction

Prickle is a sharply pointed emergence originating from multiple cellular divisions from the epidermis or cortex, which lack vasculature. It is a typical structure involved in thermoregulation and defense against multiple biotic and abiotic stresses (Kellogg et al., 2011). *Rosa roxburghii* Tratt, a Chinese perennial Rosaceae rosebush, is well-known for its high nutritional and medicinal values, but produces prickles on the surface of stem, sepals, petioles, and fruits (Wang D. J. et al., 2019; Yan et al., 2021). Its fruit prickles exhibit multiple morphological structures, including flagelliform, triangular, conical, and acicular glandular shapes at various stages (Wang D. J. et al., 2019; Li et al., 2022). However, the fruit prickles are considered an undesirable trait that damages the gardening management and injures workers. The fruit prickles of *R. roxburghii* were considered trichomes are large cells and oval in shape with large vacuoles and thin cell walls with large intercellular spaces containing abundant starch grains and plastids (Wang et al., 2021). Although the internal structure of fruit prickles has been available, precise histological observation is required for clearly deciphering the initiation and development of fruit prickles.

A fruit prickle is considered an extension or a lignified modification of the glandular trichomes, which are accompanied by deposition of phenolic compounds and development into sharp and hard appendages, thus resulting in non-glandular prickles at the late stage (Kellogg et al., 2011). Trichomes are unicellular or multicellular appendages that originate only from the protoderm of aerial epidermis cells and are diverse according to the location, morphology, and secretion of the individual plant (Serna and Martin, 2006; Wester et al., 2009). The underlying genetic and molecular mechanisms of trichome development are well understood in the model *Arabidopsis thaliana*. Furthermore, numerous genes have been previously identified through glabrous mutants and gene cloning (Walker et al., 1999; Payne et al., 2000; Zhang et al., 2003). TTG1 contains WD-40 motifs that interact with the bHLH transcription factor GLABRA3 (GL3) or ENHANCER OF GLABRA3 (EGL3) (Humphries et al., 2005). Subsequently, GL3/EGL3 interacts with the MYB transcription factor GLABRA1 (GL1) to form an MYB-bHLH-WD40 (MBW) chimera complex, which activates the downstream target gene *GLABRA2* (*GL2*). The homeodomain-leucine zipper protein GL2 finally induces trichome initiation and growth (Balkunde et al., 2011). In addition, the TTG1 protein directly binds to various bHLH proteins, which further interact with a larger of multiple MYB proteins to form the TTG1-bHLHs-MYBs complex that plays different pleiotropic roles, such as root hair formation, flavonoid biosynthesis pathway, seed pigmentation, and flowering time (Zhang et al., 2003; Chen et al., 2015). The *ttg1* mutant produced aborted trichomes and had a defective anthocyanin phenotype due to the failure of the MBW complex formation (Zhang et al., 2003). The single amino acid substitutions in the TTG1 protein could generate different phenotypes, including proanthocyanin accumulation, trichome, and root hair production in *Arabidopsis.* Besides, the amino acid changes in TTG1 altered the interaction of TTG1 with GL3 (Long and Schiefelbein, 2020).

Extensive studies have been performed mainly on stem prickles in the Rosaceae family for garden plants or cut flower production. Based on the ultrastructural observation of stem prickles of rose (*Rosa hybrida*), blackberry (*Rubus rubus*), and raspberry (*Rubus idaeus*), the well-accepted mainstream theory is that stem prickle is an outgrowth of the epidermis formed by multiple cellular divisions and it lacks vasculature thereby being biologically similarity to the trichome (Kellogg et al., 2011). Recently, using morphological studies of rose, stem prickles have been proposed to originate from multiple cells of the ground meristem beneath the protoderm that differs from the modified trichomes derived from the protoderm (Zhou et al., 2021). Based on the transcriptomic approach, homologs of the trichome candidate genes that regulate stem prickles’ development have been identified in the Rosaceae family. After screening the quantitative trait locus (QTL) region on Chr3 of a haploid rose line from *Rosa chinensis*, RC3G0244800 among them exhibited substantial similarity with *TTG2*, which acts genetically downstream of MBW while sharing the partial activity with *GL2* to initiate trichome development (Hibrand Saint-Oyant et al., 2018). The high expression of *RcGL2* triggered the differentiation of the epidermal cells to form an outgrowth of rose (Swarnkar et al., 2021). RNA-seq analysis found that the *GL1* homolog R2R3-MYB gene was downregulated in the prickle-free raspberry (Khadgi and Weber, 2020). *TTG1* expression significantly decreased in the glabrous cultivar of *Rosa chinensis* named “Basye’s Thornless” and increased in a densely prickled cultivar of *Rosa rugosa* (Feng et al., 2015; Zhou et al., 2020). The previous results were mainly based on gene expression or transcriptome analysis between prickle and prickle-less cultivars. Therefore, an in-depth investigation of the candidate gene that played in the development of fruit prickles should be made.

Some differences are observed between fruit and stem prickles in the rosaceous plants, despite similar morphology and function (Wang D. J. et al., 2019). The cells of fruit prickles contain granules, a high cytoplasm content, a range of plastids, and thin cell walls. In contrast, few organelles were observed in the cytoplasm of stem prickles cells with thick cell walls (Wang et al., 2021). Furthermore, the fruit prickles of *R. roxburghii* were distributed with spindle orientation, whereas binate prickles arose on the stem nodes (Wang D. J. et al., 2019). The potential functions and molecular mechanism of the BMW complex underlining fruit prickle are unknown. Previously, we found that *RrGL1* from *R. roxburghii* was evolutionarily similar to GL1 and positively regulated the epidermal cell fate (Huang et al., 2019). *RrEGL3* had significantly higher expression than *RrGL3* in *R. roxburghii* (Yan et al., 2021). However, *EGL3* in *Arabidopsis* had a much smaller effect than GL3 on trichome development (Bernhardt et al., 2003). Considering the ultrastructural and distributional divergence of stem and fruit prickles and the functional divergence of *RrEGL3* and *RrGL3*, it is requested to demonstrate the function and molecular mechanisms of RrTTG1 in fruit prickles of *R. roxburghii*.

In this study, we elucidated their development from floral bud to mature fruit using the morphological and histological analyses of fruit prickles. Furthermore, we studied the homologous candidate gene *TTG1* in *R. roxburghii*. qRT-PCR analysis and *in situ* hybridization revealed that *RrTTG1* was constitutively expressed and highly expressed from fruit prickle primordium to young fruit prickles. Ectopic expression of *RrTTG1* could promote trichomes formation, inhibit root hair development, and increase seed coat pigmentation and anthocyanin biosynthesis in *Arabidopsis*. The complex RrTTG1-RrEGL3-RrGL1 was detected by yeast two-hybrid (Y2H) and bimolecular fluorescence complementation (BiFC) assays. The results would lay the ground for further deciphering the regulatory mechanisms and genetic improvement of prickle-free *R. roxburghii*.

## Materials and methods

### Plant materials

*R. roxburghii* cultivar “Guinong 5” plants were planted in Guizhou Normal University (Guiyang, China, N 26^°^42.408′; E 106^°^67.353′). Stems, leaves, petioles, and sepals were collected before flowering in April. The reproductive tissues were collected from April to August, including seeds, receptacles, and fruits at 20, 40, 60, 80, and 100 days after pollination (DAP). Some of the materials for the paraffin section were stored in the FAA solution (50% ethanol, 4% formaldehyde, and 10% acetic acid). The others were frozen in liquid nitrogen immediately and stored in a freezing chamber at −80^°^C.

All seeds of *Arabidopsis* were in Columbia ecotype (Col-0) background. The plants were sterilized and plated on 1/2 MS (Murashige and Skoog) medium by keeping them in darkness at 4°C for 3 days and then transformed into an environment-controlled chamber under long-day conditions (16-h light/8-h dark) at 25°C.

### Morphological and histological observation

Materials were observed by a stereomicroscope (Leica S8 APO, Germany). The tissues were fixed in the FAA solution and then subjected to vacuum infiltrations until the tissue pieces sank. The paraffin sections were performed as previously described (Huang et al., 2016). Finally, the tissues were embedded in wax blocks and sectioned at 8 μm. The sections were viewed with an Olympus BX53 microscope (Olympus, Japan) and imaged using a SPOT FLEXTM camera (SPOT, United States).

### Gene amplification and phylogenic tree analysis

The cDNA regions were isolated by 5′ and 3′ RACE assay with the SMART™ RACE. Total RNA extracts as templates and degenerated primers of *RrTTG1* (Supplementary Table 1) were used for SMART™ RACE cDNA amplification KIT (Clontech, United States). The final amplification products were cloned into the pMD18-T vector (TaKaRa, Japan) and sequenced. For phylogenic tree analysis, multiple sequence alignment was used by ClustalW^1^ with default parameters. The alignments were submitted to MEGA 10^2^ to construct an unrooted phylogenic tree using a neighbor-joining statistical method and bootstrap analysis (1,000 replicates).

### Subcellular localization assay

The CDS of *RrTTG1* was cloned in-frame into the *Xba*I-digested pM999-eGFP plasmid, which contains an enhancer green fluorescent protein (eGFP) tag driven by CaMV 35S promoter. The constructed and empty vector plasmids were independently transformed into suspension protoplasts of *Arabidopsis* by PEG (polyethylene glycol) transformation (Yan et al., 2021). The fusion protein localization was monitored in the next 24 h. The nucleus was stained with 4′,6-diamidino-2-phenylindole (DAPI; Roche, Switzerland) at 0.1 μg/mL for 10 min. Meanwhile, the cell membrane was labeled by chloromethyl-benzamidodialkyl carbocyanine (CM-Dil, Thermo Fisher Scientific, United States) at 5 μg/mL for 15 min. The fluorescence was visualized using a laser confocal scanning microscope (SP8, Leica, Germany).

### The real-time quantitative PCR

Total RNA from the above tissues was isolated using TRIzol reagent (TaKaRa) with DNase I treatment according to the manufacturer’s instructions. After DNase treatment, RT-PCR Kit^®^ (TaKaRa) was performed according to the suppliers’ instructions using 2 μg of RNA and oligo dT-adaptor primer. The real-time quantitative PCR of *RrTTG1* was performed in a LightCycler480 instrument (Roche, Switzerland). Each reaction contained 10 μL of SYBR green PCR master mix (TaKaRa), 1.0 μL cDNA, and 200 nM primers to the final volume of 20 μL. Amplification was performed at 95°C for 5 min, 40 cycles with 95°C for 20 s, 60°C for 30 s, and 72°C for 1 min. The expression levels relative to the *RrActin* were estimated by calculating △△Ct and subsequently analyzed using 2^–△^^△^ *^Ct^* method. All samples were performed for three biological replicates.

### *In situ* hybridization

The gene-specific fragments were amplified by PCR with the ISH-F/R primer (Supplementary Table 1) and cloned into the pGM-T vector (Tiangen, China) following the manufacturer’s instructions for a DIG Northern Starter kit (Roche, Switzerland), and digoxigenin-labeled sense and antisense RNA probes were synthesized using SP6 and T7 RNA polymerase, respectively. Then, prehybridization, hybridization, washing, and detecting were performed as previously described (Yan et al., 2021).

### *Arabidopsis* transformation and phenotypic observation

*RrTTG1* was cloned into pBI121 to construct an overexpression vector *via Xba*I/*Bam*HI. Then, the constructed vector was transformed into *Agrobacterium tumefaciens* strain GV3101. The 6-week-old seedlings of *Arabidopsis* were transformed following the floral dipping method (Zhang et al., 2006). The harvested T_1_ seeds of transformed plants were sown on 1/2 MS medium containing 50 mg/L kanamycin. After kanamycin selection and RT-PCR confirmation (the primers were listed in Supplementary Table 1), the positive lines were selected, and phenotypes of transgenic plants were observed. For anthocyanin observation, 5-day-old seedlings and seeds were imaged using a Leica S8 APO stereomicroscope coupled to a Leica DC300 camera. For hair density, the number of hairs on the first 5 mm from the primary root tip of at least 30 plants per genotype was counted. Meanwhile, the number of trichomes on the first rosette leaves of 1-week-old seedlings was counted.

### Extraction and quantification of anthocyanin evaluation

The 4-week-old *Arabidopsis* seedlings were ground thoroughly in liquid nitrogen and dissolved in the extraction solution (75% H_2_O, 24% methanol, and 1% formic acid) at 4°C for 12 h to extract anthocyanins. Then, the extracts were subjected to a 10 min centrifugation at 12,000 rpm. After centrifugation, the supernatant was filtered using a 0.22 μm sterile organic filter membrane. Dried delphinidin 3,5-diglucoside chloride (Sigma-Aldrich, United States) was used to make regression equations to measure the content of anthocyanin in samples. A C18 column (4.6 × 200 mm, 5 μm, Waters Corporation, United States) was used. The mobile phase consisted of solvent A: 5% (v/v) formic acid in water and solvent B: 5% (v/v) formic acid in acetonitrile. The anthocyanin was separated by starting with 100% A with a linear gradient to 25% B over 30 min, ramping to 80% B over 2 and 3 min to re-equilibrate to initial in acetonitrile (Pourcel et al., 2010). The absorbance of the mixtures was measured at 525 nm. The anthocyanin contents were expressed as μg/100 mg of fresh weight.

### Yeast two-hybrid assay

For the Y2H analysis, the CDS of *AtGL3*, *AtEGL3*, *RrGL3*, and *RrEGL3* was amplified and ligated into pGADT7 (AD) through *Bam*HI and *Sac*I double digestion sites. Full-length CDS of *RrTTG1*, *AtTTG1*, and *RrGL1* was amplified and inserted into pGBKT7 (BD) at *Eco*RI and *Sma*I sites. Each combination of AD and BD plasmids was co-transformed into yeast strain AH109 separately using the LiAC/PEG method (Gietz and Schiestl, 2007). pGADT7-T was co-transformed with pGBKT7-53 as a positive control. pGADT7-T and pGBKT7-Lam were transformed as the negative controls (Supplementary Figure 1). All transformants were grown on SD-Trp-Leu (SD-LW), SD-Trp-Leu-Ade-His (SD-LWAH), and SD-LWAH-X-α-gal medium to detect the α-galactosidase activity of the yeast strains. The images were photographed at 5 day after incubation.

### Bimolecular fluorescence complementation assay

Full-length CDS of *AtGL3*, *AtEGL3*, *RrGL3*, and *RrEGL3* was amplified and ligated into pXY105 (cYFP) by *Bam*HI and *Sal*I sites for BiFC. Then, CDS of *RrTTG1*was amplified and inserted into pXY106 (nYFP) by *Bam*HI and *Xba*I sites. These vectors were separately transformed into *Agrobacterium* EHA105. Transformants were harvested when OD_600_ reached 0.8 and resuspended in 1/2 MS medium (supplied with 50 μM acetosyringone). The agrobacterial cells containing the cYFP fusion vector were mixed with agrobacterial cells containing the nYFP fusion vector at a 1:1 ratio. The mixture was transiently expressed in tobacco (*Nicotiana benthamiana*) leave as reported (Lee et al., 2008). The YFP fluorescence signal was visualized and detected after 48 h by using a Leica SP5X confocal microscope (Leica Co., Germany).

## Results

### The origination and development of fruit prickles in *Rosa roxburghii*

**FIGURE 1:**
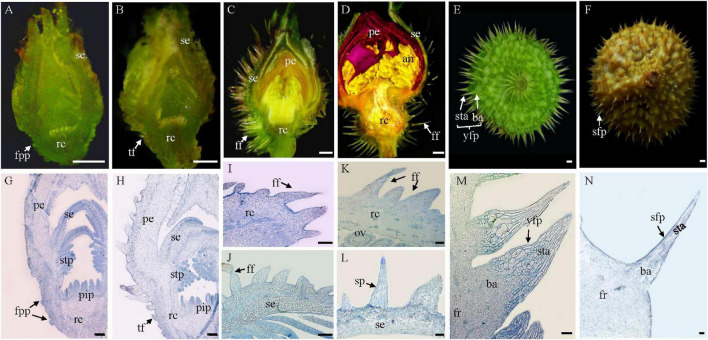
Origination and development of fruit prickles in *R. roxburghii.* **(A–F)** The macroscopic depiction of the development of fruit prickles during flower development **(A–D)** and fruit development **(E,F)**. Anatomy of fruit prickle primordium (fpp) **(G)**, triangular-shaped fpp **(H)**, flagelliform fpp of sepal **(I,J)**, flagelliform fpp of the receptacle **(K,L)**, young fruit prickle on 20 DAP fruit **(M)**, and sharp fruit prickle on 80 DAP fruit **(N)**. se, sepal; rc, receptacle; pe, petal; stp, stamen primordium; pip, pistil primordium; fpp, fruit prickle primordium; tf, triangular fpp; ff, flagelliform fpp; ov, ovule; an, anther; fr, fruit; sta, stalk; ba, base; yfp, young fruit prickle; sfp, sharp fruit prickle. **(A–F)**, bar = 500 μm; **(G–N)**, bar = 100 μm.

The mature fruit prickles are big, conical, branchless, and non-glandular in *R. roxburghii*. They usually have two types: flagelliform and acicular (Wang D. J. et al., 2019). To investigate their origination and development in *R. roxburghii*, we carried out a detailed morphological and histological investigation from the floral bud to mature fruit (Figure 1).

Stage I corresponds to fruit prickle initiation. During the floral differentiation (Figures 1A–C), fruit prickle primordia were first observed on the surface of the receptacle after the formation of the stamen and pistil primordia (Figures 1A,G). The fruit prickle primordium was a global bulge with 10–20 small ground meristematic cells (Figure 1G). Then, with the increasing number of fruit prickle primordia, their top cells gradually formed a triangular-shaped fruit prickle (Figures 1B,H). However, the initiation of the sepal prickle was a little earlier than the fruit prickle of the receptacle (Figures 1A,B,G,H).

Fruit prickle primordia exhibited continuous growth and color development during a period of rapid cell division in stage II (Figures 1C,D). The triangular-shaped prickle underwent constant cell division to form the flagelliform fruit prickle, which had no obvious base until the flower bloomed (Figures 1I–K), whereas the sepal prickle cells stopped dividing, began enlarging, and finally lignified when the flower bloomed (Figures 1J–L). Thus, the cell proliferation ability of the flagelliform fruit prickle on the receptacle may determine the width of the fruit prickle base in stage III.

After pollination, the receptacle gradually enlarged with the fruit prickle entering stage III, when it began to lignify and gradually harden (Figures 1E,F). The base cells of the fruit prickle kept dividing, with the top cells started expanding during this process to form the young fruit prickle. Each comprised a base and a stalk (Figure 1E). Their bases were composed of hundreds of spherical cells smaller than those of the stalk cells that comprised tinny cylindrical-shaped cells (Figure 1M). The top cells lignified when the fruits ripened, leading to a hard and sharp fruit prickle (Figures 1F,N). Therefore, these results indicated that the fruit prickle primordia originated from parenchyma cells of the receptacle during floral budding and subsequently transformed into hard and sharp prickles postfertilization.

### A WD40 protein *RrTTG1* is closely related to FvTTG1 and localized in the nucleus

**FIGURE 2:**
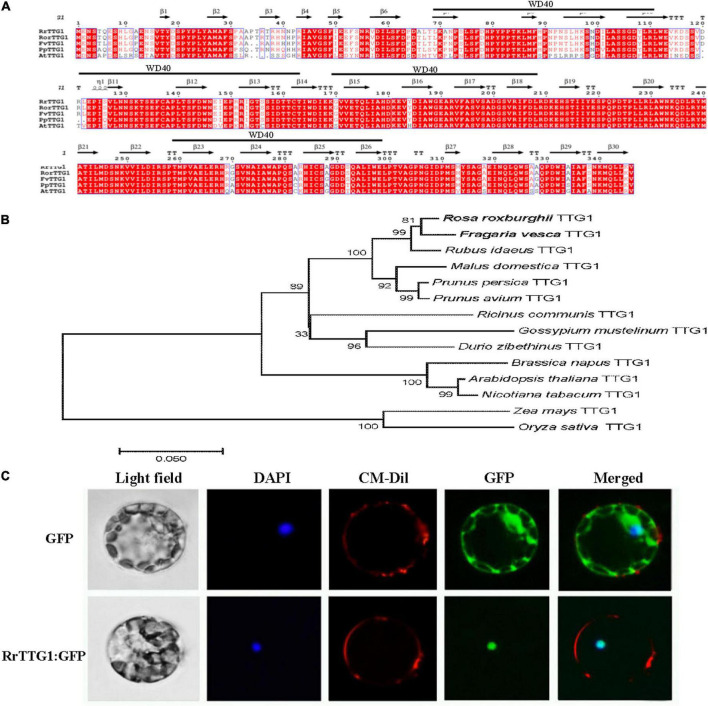
Alignment, phylogenetic, and subcellular analysis of RrTTG1. **(A)** The deduced amino acid sequence of RrTTG1 compared to TTG1 homologs from *Rosa rugosa*, *Fragaria vesca*, *Prunnus persica*, and *Arabidopsis*. Alignments were performed using Clustal W. The secondary structure was displayed above the sequences. Red shading indicated identical amino acids. The above black line represents the WD40 domain. **(B)** Phylogenetic analysis of TTG1 homologs in *R. roxburghii* and the other plants. The scale bar indicates the number of amino acid substitutions per site. GenBank accession numbers: *Fragaria vesca* TTG1 (XP_004307911.1), *Rosa rugosa* TTG1 (QCI162276.1), *Rubus idaeus* TTG1 (AEI55401.1), *Malus domestica* TTG1 (XP_008376821.1), *Prunus persica* TTG1 (ACQ65867.1), *Prunus avium* TTG1 (XP_021805418.1), *Ricinus communis* TTG1 (XP_025015182.1), *Gossypium mustelinum* TTG1 (TYI69075.1), *Durio zibethinus* TTG1 (XP_022773722.1), *Brassica napus* TTG1 (NP_001303154.1), *Arabidopsis thaliana* TTG1 (CAC10524.1), *Nicotiana tabacum* TTG1 (ACJ06978.1), *Zea mays* TTG1 (NP_001310302.1), and *Oryza sativa* TTG1 (XP_015626852.1). **(C)** RrTTG1 was localized in the nucleus. Protoplasts from *Arabidopsis* were transiently transformed with an 35S:RrTTG1-GFP or an empty vector. The empty 35S:GFP vector was used as the control. GFP, green fluorescence protein; DAPI, 4′, 6-diamino-2-phenylindol; CM-Dil, chloromethyl-benzamidodialkyl carbocyanine. Bars = 5 μm.

Since the WD40-repeat protein TTG1 functions as a critical positive regulator of trichome determined in *Arabidopsis*, a detailed analysis of TTG1 homolog in *R. roxburghii* might help us to understand the molecular mechanism of fruit prickles formation. The full-length cDNA of *RrTTG1* contains an open reading frame (ORF) of 1,041 bp that encoded a polypeptide of 346 amino acids. RrTTG1 harbored four conserved WD40 repeats, with each repeat containing nearly 44 amino acid tandems (sites at 70–113 aa, 121–166 aa, 169–207 aa, and 258–298 aa). RrTTG1 aligned with its TTG1 orthologs in woodland strawberry (*Fragaria vesca*), rose (*Rosa rugosa*), peach (*Prunnus persica*), and *Arabidopsis* (Figure 2A). RrTTG1 showed over 95% similarity with its homologs of woodland strawberry and rose. However, we observed slightly less similarity to TTG1 which showed 79% identity.

Phylogenetic analysis confirmed that RrTTG1 clusters together with the homologs from the Rosaceae family, thus displaying the closest relationship with TTG1 homologs from woodland strawberry and rose, and we also identified additional closely related sequences in homologs of raspberry and apple (*Malus domestica*) (Figure 2B). Furthermore, we also found that homologs of dicotyledonous plants, including castor beans (*Ricinus communis*), cotton (*Gossypium mustelinum*), and durian (*Durio zibethinus*), were also clustered. However, those of the homologous monocot sequences ZmTTG1and OsTTG1 were more distantly related (Figure 2B). To examine the detailed subcellular localization of RrTTG1, the fused protein RrTTG1: GFP was transformed into *Arabidopsis* protoplasts. DAPI and CM-Dil staining showed exclusively nuclear and cell membrane distributions, respectively (Figure 2C). Under the induction of 405 nm light, the protoplasts transformed with 35S:GFP presented the green fluorescent signals in the whole cell, including the membrane, cytoplasm, and nucleus. When the protoplast was transformed with 35S:RrTTG1:GFP, the GFP signals were only observed in the nucleus, similarly at the same site as DAPI, inferring that RrTTG1 was localized in the nucleus and may function as a transcription factor.

### *RrTTG1* was highly expressed during the division and expansion phases of fruits prickles

**FIGURE 3:**
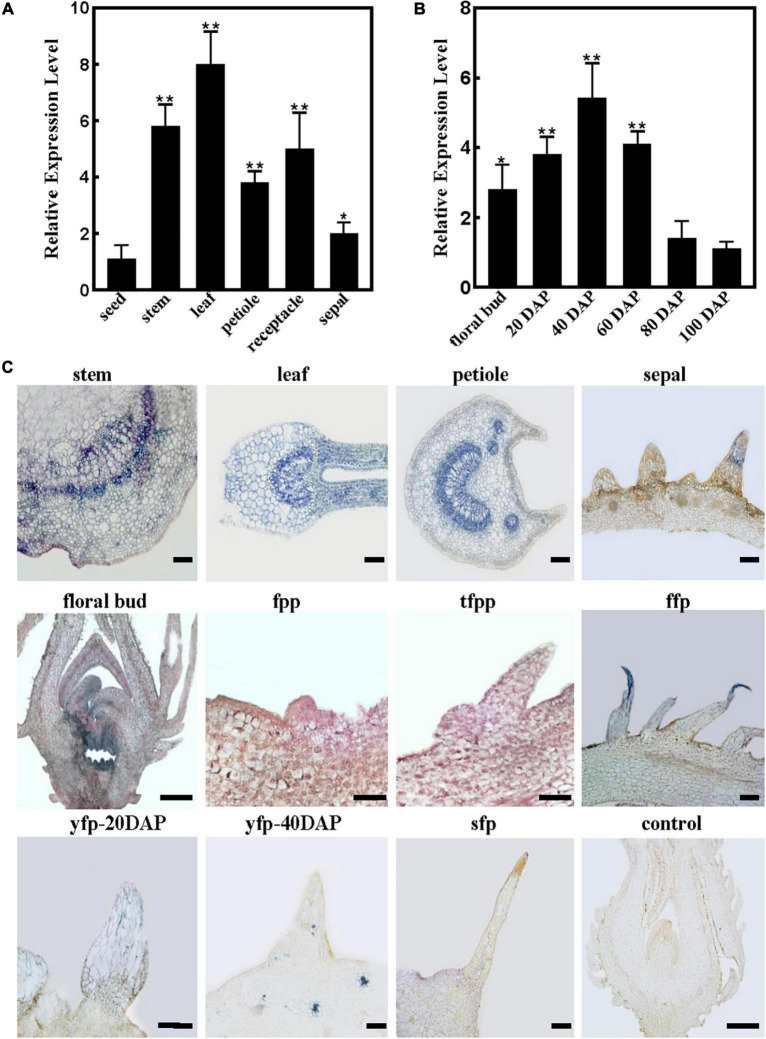
*RrTTG1* was expressed constitutively and highly at the early stage of fruit prickles formation. **(A,B)** qRT-PCR analysis of *RrTTG1* expression in various tissues **(A)** and fruits at different development stages **(B)**. Values are presented as means ± SD (*n* = 3) and analyzed using one-way ANOVA (**p < 0.01, *p < 0.05). Primers are listed in Supplementary Table 1. **(C)** *In situ* hybridization analysis of the expression pattern of *RrTTG1.* The expression of *RrTTG1* in the young stem, young leaf, petiole, sepal, floral bud, fruit prickle primordium (fpp), triangular-shaped fpp (tfpp), flagelliform fruit prickle (ffp), young fruit prickle on 20 DAP fruit (yfp-20 DAP), 40 DAP fruit (yfp-40 DAP), and 80 DAP fruit (sfp) was detected. The paraffin sections with sense RNA probes of *RrTTG1* were used as the control. Bar = 200 μm.

We selected various tissues to examine the expression patterns of *RrTTG1* and found that it was constitutively expressed in young leaf, stem, petiole, sepal, and receptacle, whereas being low in seeds in *R. roxburghii* (Figure 3A). Moreover, the expression during fruit development was detected. We found that *RrTTG1* was highly expressed in the fruits at the early stages and finally peaked at 40 DAP (Figure 3B). The transcript level was significantly reduced at 80 DAP when fruits were in the turning stage, and it reached the lowest at 100 DAP when fruits fully ripened (Figure 3B). Thus, these results mainly demonstrated that *RrTTG1* was essential in the early stage of fruit prickle development.

To further identify the spatial and temporal expression patterns of *RrTTG1* during fruit prickle development, *in situ* hybridization was performed (Figure 3C). The sense probe showed no detectable signal as the control. Despite the different arrangements of xylem and phloem cell types in the various organs, the cells in vascular bundles, cambium, and parenchyma cells of the young stem, young leaf, and petiole showed a much higher *RrTTG1* transcript signal, which was consistent with the qRT-PCR results (Figure 3C). We also detected strong visible signals in the fruit prickle primordia and triangular-shaped fruit prickle primordia, especially the top cells at the flagelliform fruit prickle, which indicated the highly abundant transcript of *RrTTG1* being involved in the prickle initiation (Figure 3C). *RrTTG1* continued to be expressed highly in the parenchymal cells of fruit prickles on 20 DAP fruit. In the 40 DAP fruit, despite the high expression of *RrTTG1* in the vascular bundles of fruit fresh, its expression decreased in the cells of fruit prickle. Corresponding with the low *RrTTG1* expression in fruits at 80 DAP, there was no obvious signal in the sharp fruit prickle (Figure 3C). Therefore, the results indicated that *RrTTG1* likely enhanced fruit prickle differentiation and enlargement.

### *RrTTG1* decreased the number of root hairs and enhanced the anthocyanin content

**FIGURE 4:**
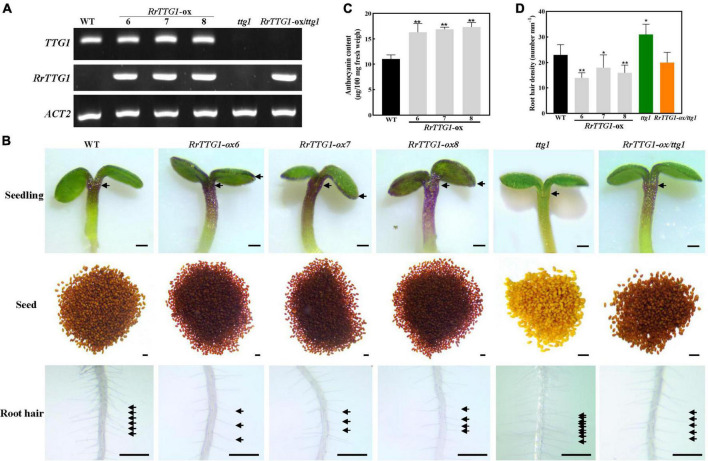
*RrTTG1* promoted anthocyanin content and inhibited root hair formation. **(A)** Expression of *TTG1* and *RrTTG1* in WT (Col-0), *ttg1* mutant, and transgenic plants (*RrTTG1*-ox and *RrTTG1*-ox/*ttg1*). RT-PCR was used to detect the expression of *TTG1* and *RrTTG1*, and *ACT2* was used as a control. Primers are listed in Supplementary Table 1. **(B)** Ectopic expression of *RrTTG1* could promote anthocyanin content and inhibit root hair formation in *Arabidopsis*. The seeds and 5-day-old seedlings were collected for anthocyanidin observation. Root hair located 5 mm above the root tip was observed, bar = 1 mm. **(C)** Ectopic expression of *RrTTG1* could promote anthocyanin content. The anthocyanin contents of 4-week-old WT and *RrTTG1-ox* transgenic plants were measured by HPLC analysis. **(D)** Ectopic expression of *RrTTG1* could reduce the root hair density. The number of root hairs per mm (root hair density) of WT, *ttg1* mutant, and transgenic plants (*RrTTG1*-ox and *RrTTG1*-ox/*ttg1*) was counted (*n* = 30). Data are mean ± SD and analyzed using one-way ANOVA (**p < 0.01, *p < 0.05).

Due to no feasible transformation system for *R. roxburghii*, we ectopically expressed *RrTTG1* in the *Arabidopsis* plants to explore its function. The transcript of *RrTTG1* was detectable in the seedlings of transgenic plants, including three *RrTTG1-ox* lines and *RrTTG1-ox/ttg1* plants, inferring that *RrTTG1* was expressed in *Arabidopsis* (Figure 4A). The colors of hypocotyl and cotyledon of the seedlings in the *RrTTG1-ox* plants were more intense than wild-type (WT) (Figure 4B). The results of anthocyanin quantification showed a significant increase in *RrTTG1-ox* plants. The anthocyanin contents of three transgenic plants increased by 48.19, 53.64, and 57.45% compared to the WT, respectively (Figure 4C). Moreover, pigmentation of the seed coat endothelium was more condensed in *RrTTG1-ox* plants, while its color was less brown in WT, thereby suggesting that *RrTTG1* could enhance the anthocyanin accumulation. Consistently, ectopic expression of *RrTTG1* could rescue the pigmentation phenotype of the *ttg1* mutant in *Arabidopsis* (Figure 4B).

We analyzed the effect of the transgene on root hair development. WT plants showed well-spaced root hairs, whereas the roots of *RrTTG1-ox* plants displayed sparsely spaced irregularly growing root hairs (Figure 4D). The *RrTTG1-ox* plants showed an average 30.43% decrease of root hair density compared to the WT. In addition, the *ttg1* mutant produced approximately 1.35-fold of root hairs in WT root, while the *RrTTG1-ox/ttg1* transgenic plants exhibited a near-normal root hair pattern (Figure 4D), thereby deducing that *RrTTG1* substantially inhibited root hair formation.

### *RrTTG1* promoted the trichome number in *Arabidopsis*

**FIGURE 5:**
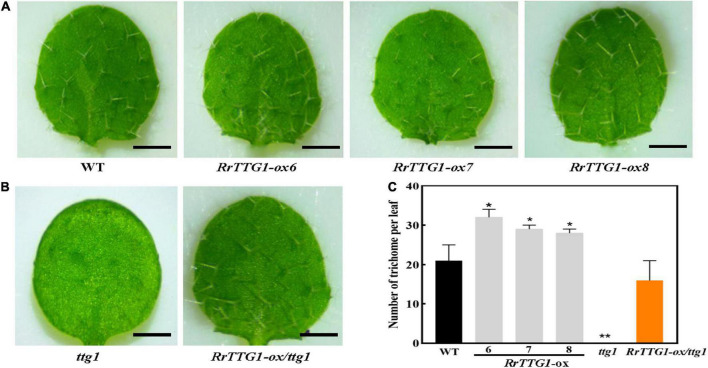
*RrTTG1* promoted trichome formation in *Arabidopsis.* **(A)** Ectopic expression of *RrTTG1* could increase the trichome number in *Arabidopsis*. Leaf trichome phenotypes from the first two rosette leaves of 1-week-old soil-grown seedlings of the WT (Col-0) and three *RrTTG1-ox* transgenic plants were compared, bar = 1 cm. **(B)** *RrTTG1* complemented the glabrous phenotype of *ttg1* mutant. Leaf trichome phenotypes from the first two rosette leaves of one-week-old soil-grown seedlings of the *ttg1* and three *RrTTG1*-ox*/ttg1* transgenic plants were compared, bar = 1 cm. **(C)** Comparison of the trichome number per leaf in the WT, *ttg1* mutant, and transgenic plants (*RrTTG1*-ox and *RrTTG1*-ox/*ttg1*). Values are means ± SD (*n* = 30) and analyzed using one-way ANOVA (**p < 0.01, *p < 0.05).

To test the effect of *RrTTG1* on trichome development, we observed the trichome phenotype in transgenic plants. We noticed that *RrTTG1-ox Arabidopsis* plants had a significantly greater number of trichomes per leaf than that of WT, and their trichome number increased by about half, finally reaching 52.38, 38.10, and 33.33%, respectively (Figures 5A,C). Undoubtedly, the trichome was absent in the *ttg1* mutant. *RrTTG1* was able to functionally complement the glabrous phenotype of the *ttg1* mutant (Figures 5B,C). Our results indicated that *RrTTG1* was functionally homologous to the *TTG1* regulating trichome development, thus inferring that *RrTTG1* might function as TTG1 homologs in the Rosaceae family to modulate the fruit prickles development of *R. roxburghii*.

### The *RrTTG1* only interacted with RrEGL3 to form an MYB-bHLH-WD40 complex in *Rosa roxburghii*

**FIGURE 6:**
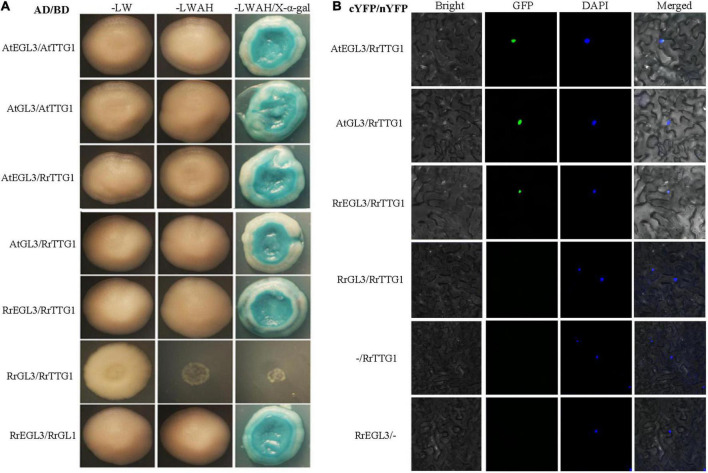
RrTTG1 only interacted with RrEGL3 to form the RrTTG1-RrEGL3-RrGL1 complex. **(A)** Y2H assay testing the interaction between RrTTG1 and GL3/EGL3/RrGL3/RrEGL3. Each combination of AD and BD plasmids was co-transformed into yeast strain AH109 separately, and all transformants were grown on SD-Trp-Leu (SD-LW), SD-Trp-Leu-Ade-His (SD-LWAH), and SD-LWAH-X-α-gal medium. The images were photographed at 5 day after incubation. All the controls were displayed in Supplementary Figure 1. **(B)** BiFC assay testing the interactions between RrTTG1 and GL3/EGL3/RrGL3/RrEGL3. Each combination of cYFP and nYFP was transiently expressed in *Nicotiana benthamiana* leave, and the YFP fluorescence signal was visualized and photographed after 48 h.

Since TTG1 interacted with the bHLH transcription factor GL3 or EGL3 to influence the trichome number and anthocyanin content in *Arabidopsis*, we performed the Y2H assay to detect the interaction partner proteins of RrTTG1. The combinations of empty vector (pGADT7 or pGBKT7) with all the tested proteins were also performed, and no self-activation was detected (Supplementary Figure 1). Y2H result showed that RrTTG1 could both directly interact with GL3 or EGL3 like TTG1 in yeast, thereby implying that RrTTG1 was evolutionarily similar to the TTG1. Notably, we found that RrTTG1 failed to bind to RrGL3 and only interacted with RrEGL3 (Figure 6A). Such interaction was also confirmed by the BiFC approach in *Nicotiana benthamiana*. The fluorescence signals revealed that RrTTG1 positively interacted with EGL3 or GL3 from *Arabidopsis*. Therefore, we also showed the positive interaction between RrTTG1 and RrEGL3. However, we did not detect any fluorescence signal from the combination of RrTTG1 and RrGL3, thus suggesting no direct interaction between RrGL3 and RrTTG1 (Figure 6B).

**FIGURE 7:**
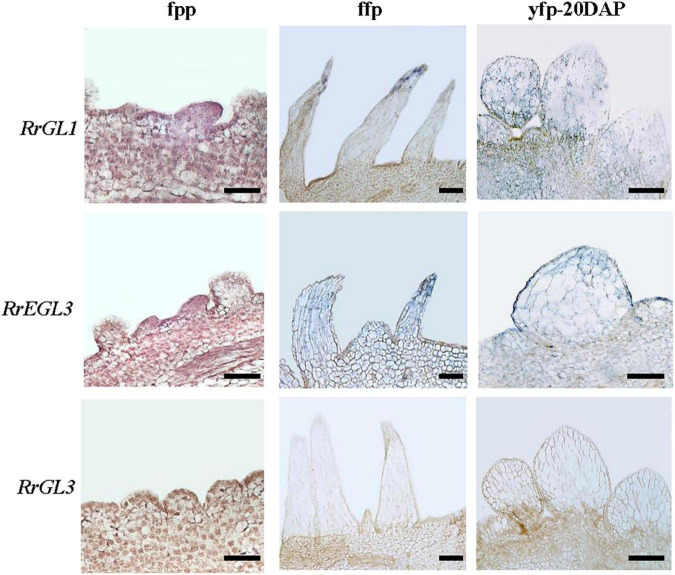
*RrGL1* and *RrEGL3* highly expressed at the early stage of fruit prickles formation. *In situ* hybridization testing the expression pattern of *RrGL1*, *RrEGL3*, and *RrGL3* in fruit prickle primordium (ffp), flagelliform fruit prickle (ffp), and young fruit prickle on 20 DAP fruit (yfp-20 DAP). Bar = 200 μm.

As *RrTTG1* was highly expressed in the initiation of fruit prickle formation, to investigate whether its partner proteins have the same expression pattern, we explored the expressions of *RrGL1*, *RrGL3*, and *RrEGL3* from fruit prickle primordia to young fruit prickle (Figure 7). The transcripts of *RrGL1* and *RrEGL3* having higher abundance were localized in the global bulges of fruit prickle primordia on the receptacle. *RrGL1* and *RrEGL3* were highly expressed in the flagelliform fruit prickle, especially *RrEGL3* which was essential in the differentiation of fruit prickle primordia. Moreover, when the cells at the base began to enlarge, we found strong signals of both *RrGL1* and *RrEGL3* in the young fruit prickles that were derived from flagelliform fruit prickle, while no visible signal of *RrGL3* was detected (Figure 7). These results showed that RrGL1 and RrEGL3, the partner proteins of RrTTG1, are also highly expressed at the early stage of fruit prickle formation. Therefore, such an expression pattern of the RrTTG1-RrEGL3-RrGL1 complex suggested it played a substantial role during the prickle primordium initiation and expansion of fruit prickles.

## Discussion

### The fruit prickle was derived from the ground meristem beneath the protoderm of the receptacle

Prickles are previously proposed as extended and deformed trichomes originating from protodermal cells (Hibrand Saint-Oyant et al., 2018), while a recent study proved that stem prickle was not modified trichome due to its initiation from the ground meristem beneath the protoderm (Zhou et al., 2021). The fruit prickles are similar to the trichomes based on morphological and anatomical studies. Both lack vascular bundles and are effective deterrents against herbivores (Papatheodorou et al., 2020). Regarding differences, trichomes comprise non-glandular, branched, and unicellular cells originating from the protoderm (Serna and Martin, 2006). However, fruit prickles of *R. roxburghii* are derived from multiple cellular divisions along with lignification, thereby leading to sharp fruit prickles (Figure 1). Likewise, the prickles of eggplant were also shown to be multicellular and lignified (Zhang et al., 2021). Furthermore, through the histological analysis, the fruit prickle primordium of *R. roxburghii* with a slight global bulge of 40–70 spherical cells originated from the ground meristem beneath the protoderm of the receptacle, which is composed of parenchyma cells (Figure 1), supporting the divergence of fruit prickle and trichome. Understanding the histological aspect of prickles will benefit the fruit’s processing and harvesting that could be extended to other plant species.

Prickles are present on organs other than the fruit, such as stems, petioles, and pedicels. The prickles on the surface of fruit were found only in some species. Thus, little is known about the development of fruit prickles, whereas stem prickles have been made in numerous studies for cut rose production and gardening (Feng et al., 2015; Khadgi and Weber, 2020). The stem prickles of rose develop from multiple cells of the ground meristem beneath the protoderm of the stem apex. We found that fruit prickles also originated from the ground meristem beneath the protoderm of the receptacle, which is the axis of floral organs attached in botany. Nevertheless, we found a significantly divergent development between fruit and stem prickles. The base cells of stem prickles underwent one rapid proliferation and gradually stopped (Zhou et al., 2021). However, the cells of fruit prickle primordium underwent the first rapid proliferation to form flagelliform-shaped fruit prickle and stopped before flowering. The base cells of flagelliform-shaped fruit prickle underwent the second proliferation, and the top cells began to elongate after pollination (Figure 1). As we know, the cells of mature fruit prickles contain granules, a high cytoplasm content, a range of plastids, and thin cell walls. In contrast, few organelles were observed in the cytoplasm of stem prickles cells with thick cell walls (Wang et al., 2021). Thereby, the developmental discrepancy may underlie the divergence in shape, distribution, and internal structure between stem prickles and fruit prickles.

### Functional conserved RrTTG1 might modulate the formation of fruit prickle in *Rosa roxburghii*

The RrTTG1 protein has four WD40-repeat domains, which act as a scaffold for various protein–protein interactions for regulating the transcriptional processes (Stirnimann et al., 2010). We found that the TTG1 homologs from *R. roxburghii* and other dicotyledonous plants were clustered in the same branch (Figure 2). The nuclear localization and constitutive expression pattern of the RrTTG1 were consistent with that of TTG1, thereby suggesting that RrTTG1 may act as a transcription factor akin to TTG1 and have conserved functions (Zhao et al., 2008). TTG1 regulated epidermal cell fate specification, including trichome development in *Arabidopsis* (Payne et al., 2000; Lin and Schiefelbein, 2001). Trichome has served as an excellent model to study the molecular mechanism of prickle or other appendages of some plants lacking the genetic transformation system (Liu et al., 2016). Ectopic expression of *RrTTG1* could increase the trichome number in *Arabidopsis* and rescue the glabrous phenotype in the *ttg1* mutant (Figures 5, 6). This might be attributed to the conserved ability of *RrTTG1* positively to interact with GL3 and EGL3 (Figure 6). Furthermore, *RrTTG1* is highly expressed in the early stages of fruit prickles from fruit prickle primordium to young fruit prickles on 40 DAP fruits (Figure 3), which corresponds with *TTG1* in the rosaceous plants is highly expressed in apical meristems or at the transition stages when cells differentiated to form stem prickles (Swarnkar et al., 2021; Zhong et al., 2021). Thus, the results demonstrated that *RrTTG1* was homologous to the functions of *TTG1* in *Arabidopsis* and might modulate the formation of fruit prickle.

### *RrTTG1* might function in fruit prickles formation through the RrTTG1-RrEGL3-RrGL1 complex in *Rosa roxburghii*

TTG1 is a central regulator by forming different MBW complexes to influence the development of appendages, including trichome, spine, prickle, and fiber (Zhang et al., 2003; Humphries et al., 2005). In *Arabidopsis*, TTG1 interacts with GL3 or EGL3 to regulate multiple processes in plants, such as trichome formation, anthocyanin production, and seed coat pigmentation, and negatively regulates root hair formation (Tian and Wang, 2020). Interestingly, *GL3* plays a positive and critical role in trichome branching rather than *EGL3* in *Arabidopsis* (Shen et al., 2006; Wang Z. et al., 2019). *CsTTG1* is also an important regulator of fruit spine and wart formation in cucumber (*Cucumis sativus*) (Cui et al., 2016; Zhang et al., 2016). Regarding rice (*Oryza sativa*), only OsGL3B can interact with OsTTG1 to form the MBW complex to promote trichomes formation (Zheng et al., 2021). Similarly, SlTTG1 only interacts with SlEGL3 to regulate the trichomes formation in tomatoes (*Solanum lycopersicum*) (Tominaga-Wada et al., 2017). In our results, despite the conserved interaction of RrTTG1 with EGL3 or GL3, RrTTG1 only interacted with RrEGL3 and failed to bind RrGL3 (Figure 6). Furthermore, the expression of *RrEGL3* was significantly higher than *RrGL3* (Yan et al., 2021). Taken together, only RrEGL3 can interact with RrTTG1 to form an MBW complex in *R. roxburghii*.

The failure of RrTTG1 to interact with RrGL3 might attribute to the loss of function of RrTTG1 or RrGL3. The amino acid substitutions (S197F and L339F) of TTG1 could reduce its ability to interact with GL3 in *Arabidopsis* (Long and Schiefelbein, 2020). However, RrTTG1 shared the same amino acids with TTG1 at these two sites, indicting its conserved function of interacting with GL3 and fully restoring the deficient phenotype of the *ttg1* mutant (Figures 4, 5). *SlGL3* was found to lose its function of regulating trichome development (Wada et al., 2014). Similarly, a single amino acid substitution in the *GL3* homologous gene *MYC1* in *Arabidopsis* led to trichome pattern defects *via* the failure to interact with TTG1 (Zhao et al., 2012). The previous study showed that *RrGL3* transcripts were absent during fruit development, and the ectopic expression of *RrGL3* did not influence trichome patterning in *Arabidopsis* (Yan et al., 2021). Thus, the aborting of the RrTTG1-RrGL3 interaction is mainly attributed to the loss of function of RrGL3. By the way, the failure of the interaction of RrTTG1 with RrGL3 helped develop the non-branch fruit prickle in *R. roxburghii*.

### A model of *RrTTG1* function in the initiation and expansion of fruit prickles in *Rosa roxburghii*

**FIGURE 8:**
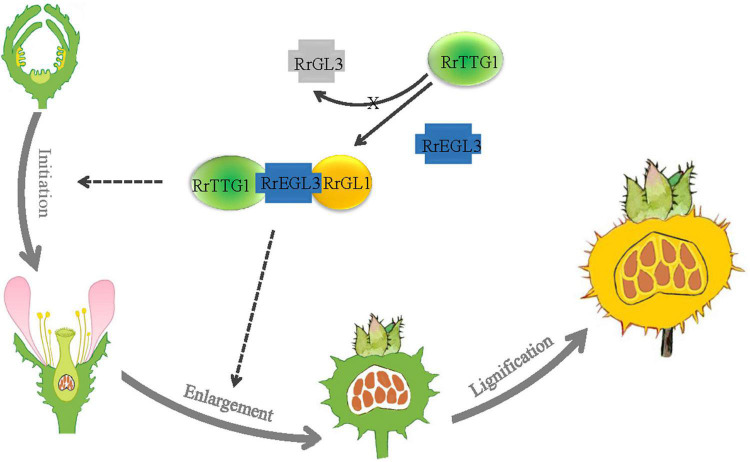
Model of RrTTG1 function in fruit prickles formation in *R. roxburghii*. Fruit prickles’ formation undergoes three stages: initiation, enlargement, and lignification. RrTTG1 only interacts with RrEGL3 to form the RrTTG1-RrEGL3-RrGL1 complex and fails to interact with RrGL3. The RrTTG1-RrEGL3-RrGL1 are highly expressed from fruit prickle primordia to young fruit prickle. Thus, RrTTG1-RrEGL3-RrGL1 might modulate the primordia initiation and enlargement of fruit prickles in *R. roxburghii*. The lines and dotted lines represent the results and speculations, respectively.

The model of RrTTG1 function in the development of fruit prickles in *R. roxburghii* was shown (Figure 8). Fruit prickles’ formation undergoes three stages: initiation, enlargement, and lignification. The initiation stage corresponds to the occurrence of the fruit prickle primordium. The primordium derives from the meristem beneath the protoderm and forms the flagelliform trichome on the surfaces of the receptacle until the flower blooms. After pollination, with the receptacle gradually enlarging, the flagelliform prickles continue to grow *via* cell division and expansion. The lignification stage is defined as the mature stage when the hard and sharp fruit prickles have formed on the surface of the fruit. RrTTG1 only interacts with the bHLH transcription factor RrEGL3 to form an MBW complex (RrTTG1-RrEGL3-RrGL1) and fails to interact with RrGL3. The RrTTG1-RrEGL3-RrGL1 is highly expressed from fruit prickle primordia to young fruit prickle. Thus, RrTTG1-RrEGL3-RrGL1 might modulate the primordia initiation and enlargement of fruit prickles in *R. roxburghii*.

## Data availability statement

The datasets presented in this study can be found in online repositories. The names of the repository/repositories and accession number(s) can be found in the NCBI repository, accession number: ON381945.

## Author contributions

XH: writing, visualization, and editing. PY: formal analysis and investigation. YL, YJ, and QL: conceptualization and funding acquisition. YY and HY: project administration and supervision. All authors contributed to the article and approved the submitted version.

## Footnotes

1
https://www.genome.jp/tools-bin/clustalw

2
https://www.megasoftware.net/

## Supplementary material

Supplementary Figure 1 Control experiment of yeast two-hybrid assay. The combinations of pGADT7 and pGBKT7-Lam, as well as pGADT7 and pGBKT7-53, were used as negative and positive control, respectively. Meanwhile, the combinations of empty vector (pGADT7 or pGBKT7) with all the tested proteins were also performed, and no self-activation was detected.
