# Supplementary material for: RrTTG1 promotes fruit prickle development through an MBW complex in Rosa roxburghii
Source: Front Plant Sci. 2022 Aug 29;13:939270. doi: 10.3389/fpls.2022.939270 (PMC9465040; doi:10.3389/fpls.2022.939270)
Supplement: Supplementary file 1 [file Table_1.DOCX]

**Table S1.** **The Primers used for amplification and functions of *RrTTG1*.**

| **Name** | **Application** | **Primer sequence** |  |  |
| --- | --- | --- | --- | --- |
| *RrTTG1*-GSP1 | 5’-RACE | CGGCAGCTCCCAAATAAGCGCCT |  |  |
| *RrTTG1*-GSP2 | 3’-RACE | TACGCCATGGCGTTTTCCCCG |  |  |
| PM999-*RrTTG1*-F | subcellular | GCTCTAGAATGGAGAACTCGACTCAAGAA |  |  |
| PM999-*RrTTG1*-R | subcellular | GCTCTAGAAACCTTCAAAAGCTGCATCTT |  |  |
| *RrTTG1*-F | qRT-PCR/ISH | AGGGTTTTCGCCTCCGTCTCC |  |  |
| *RrTTG1*-R | qRT-PCR/ISH | CTTGTTACTATCCATCAGAAT |  |  |
| *RrGL3*-F | ISH | ATCAACTATGCTGTGTCGAAGAAC |  |  |
| *RrGL3*-R | ISH | AGTATCTAAGTGGAGACTGCTTGT |  |  |
| *RrEGL3*-F | ISH | TTGCCAGGAAGAGCATTAG |  |  |
| *RrEGL3*-R | ISH | CTCAACAACACCTCCAAGA |  |  |
| *RrGL1*-F | ISH | ACTACTTCCTCAGACCAACAAG |  | |
| *RrGL1*-R | ISH | TGCTGCTGCTTCACCATAG |  |  |
| *Rractin*-F | qRT-PCR | AATCTTGACAGAGCGTGGTTAC |  |  |
| *Rractin*-R | qRT-PCR | GGATGGCTGGAAGAGGACTT |  |  |
| *TTG1*-F | qRT-PCR | TACAACAACCGCATCGACAT |  |  |
| *TTG1*-R | qRT-PCR | GAGATCTCCGGAGGAAGGAC |  |  |
| *actin*-2-F (At3g18780) | qRT-PCR | GCTGAGGCTGATGATATTCAAC |  |  |
| *actin*-2-R (At3g18780) | qRT-PCR | CGTACAAGGAGAGAACAGCTT |  |  |
| pBI121-*RrTTG1*- F | overexpression | GGACTCTAGAATGGAGAACTCGACTCAAGAA | |  |
| pBI121-*RrTTG1*- R | overexpression | CCGGGGATCCAACCTTCAAAAGCTGCATCTT | |  |
| PGADT7-*AtGL3*-F | Y2H | CGGGATCCATGGCTACCGGACAAAACAGA | |  |
| PGADT7-*AtGL3*-R | Y2H | CGAGCTCTCAACAGATCCATGCAACCCT | |  |
| PGADT7-*AtEGL3*-F | Y2H | CGGGATCCATGGCAACCGGAGAAAACAGA | |  |
| PGADT7-*AtEGL3*-R | Y2H | CGAGCTCTTAACATATCCATGCAACCCT | |  |
| PGADT7-*RrGL3*-F | Y2H | CGGGATCCATGGCCAATGGGACTCAAATC | |  |
| PGADT7-*RrGL3*-R | Y2H | CGAGCTCACACTTACCAGCAATTTTCCA | |  |
| PGADT7-*RrEGL3*-F | Y2H | CGGGATCCATGGGTACTAGGCTCCAGAAC | |  |
| PGADT7-*RrEGL3*-R | Y2H | CGAGCTCACAGTTCCTAGCGATTCTCTG | |  |
| PGBKT7*-AtTTG1*-F | Y2H | CGGGATCCATGGATAATTCAGCTCCAGA | |  |
| PGBKT7*-AtTTG1*-R | Y2H | CGAGCTCACGTCGAGGAATCTCAAACT | |  |
| PGBKT7*-RrTTG1*-F | Y2H | CGGAATTCATGGAGAACTCGACTCAAGAA | |  |
| PGBKT7*-RrTTG1*-R | Y2H | TCCCCCGGGAACCTTCAAAAGCTGCATCTT | |  |
| PGBKT7*-RrGL1*-F | Y2H | CGGAATTCATGGAAGGTGGTGGAAGGAAT | |  |
| PGBKT7*-RrGL1*-R | Y2H | TCCCCCGGGGAAGCCGATATCCTCAAAA | |  |
| pXY106-*RrTTG1*-F | BIFC | ACAACATCGAGGACGCCGGCGGATCCATGGAGAACTCGACTCAAGAA | |  |
| pXY106-*RrTTG1*-R | BIFC | TACGAACGAAAGCTCTGCAGTCTAGAAACCTTCAAAAGCTGCATCTT | |  |
| pXY105-*RrGL3*-F | BIFC | ACGAGCTGTACAAGGCCGGCGGATCCATGGCCAATGGGACTCAAATC | |  |
| pXY105-*RrGL3-*R | BIFC | TACGAACGAAAGCTCTGCAGGTCGACACACTTACCAGCAATTTTCCA | |  |
| pXY105-*RrEGL3*-F | BIFC | ACGAGCTGTACAAGGCCGGCGGATCCATGGGTACTAGGCTCCAGAAC | |  |
| pXY105-*RrEGL3*-R | BIFC | TACGAACGAAAGCTCTGCAGGTCGACACAGTTCCTAGCGATTCTCTG | |  |
| pXY105-*AtGL3*-F | BIFC | ACGAGCTGTACAAGGCCGGCGGATCCATGGCTACCGGACAAAACAGA | |  |
| pXY105-*AtGL3*-R | BIFC | TACGAACGAAAGCTCTGCAGGTCGACTCAACAGATCCATGCAACCCT | |  |
| pXY105-*AtEGL3*-F | BIFC | ACGAGCTGTACAAGGCCGGCGGATCCATGGCAACCGGAGAAAACAGA | |  |
| pXY105-*AtEGL3*-R | BIFC | TACGAACGAAAGCTCTGCAGGTCGACTTAACATATCCATGCAACCCT | |  |
