# Supplementary figures and images for: RrTTG1 promotes fruit prickle development through an MBW complex in Rosa roxburghii
Source: Front Plant Sci. 2022 Aug 29;13:939270. doi: 10.3389/fpls.2022.939270 (PMC9465040; doi:10.3389/fpls.2022.939270)

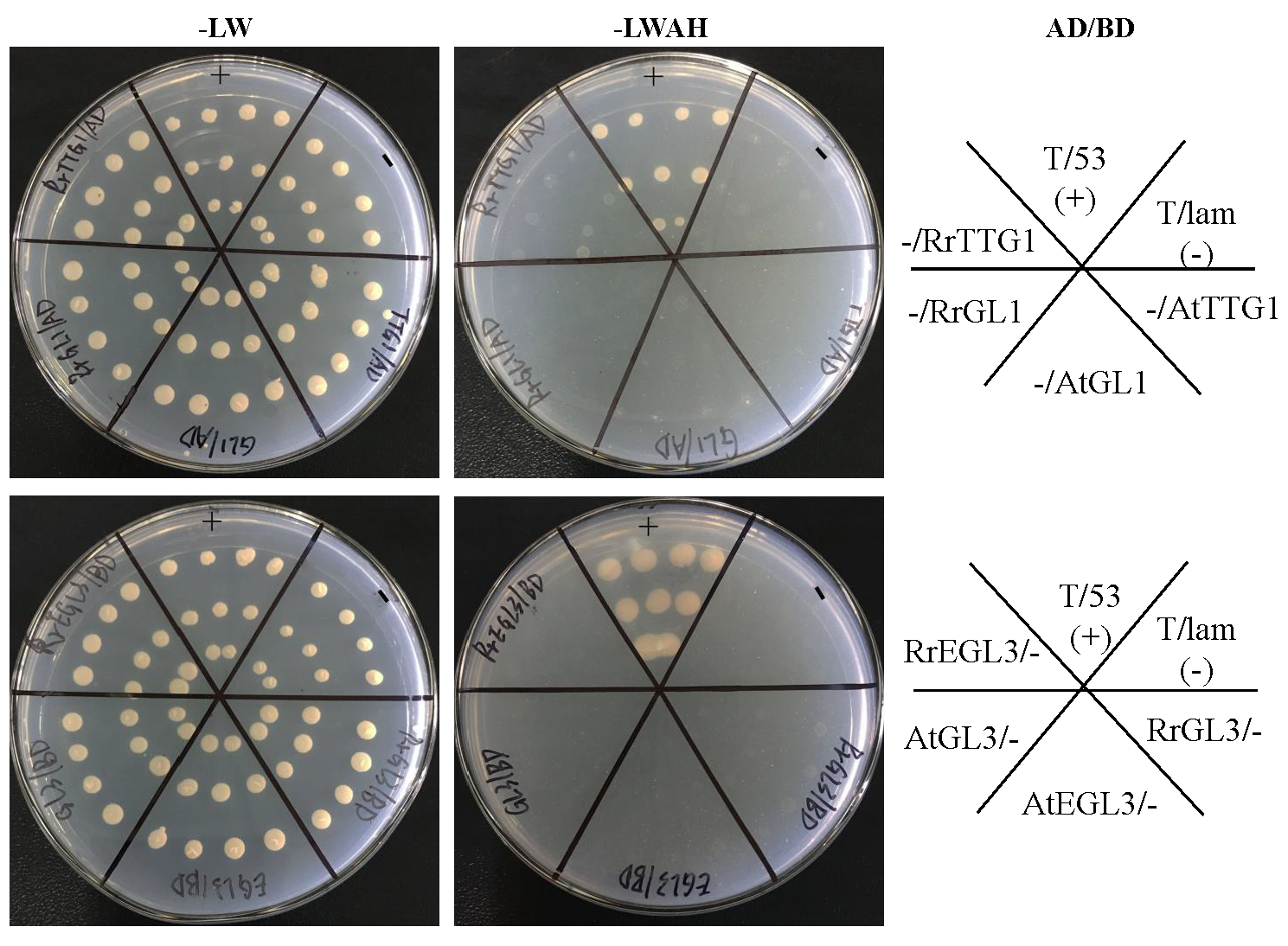

Supplement: Supplementary Figure 1 — Control experiment of yeast two-hybrid assay. The combinations of pGADT7 and pGBKT7-Lam, as well as pGADT7 and pGBKT7-53, were used as negative and positive control, respectively. Meanwhile, the combinations of empty vector (pGADT7 or pGBKT7) with all the tested proteins were also performed, and no self-activation was detected. [file Image_1.TIF]
